# MYFix: Automated Fixation Annotation of Eye-Tracking Videos

**DOI:** 10.3390/s24092666

**Published:** 2024-04-23

**Authors:** Negar Alinaghi, Samuel Hollendonner, Ioannis Giannopoulos

**Affiliations:** Research Division Geoinformation, Vienna University of Technology, Wiedner Hauptstraße 8/E120, 1040 Vienna, Austria; samuel.hollendonner@geo.tuwien.ac.at (S.H.); igiannopoulos@geo.tuwien.ac.at (I.G.)

**Keywords:** automatic fixation annotation, object detection, semantic segmentation, outdoor mobile eye-tracking

## Abstract

In mobile eye-tracking research, the automatic annotation of fixation points is an important yet difficult task, especially in varied and dynamic environments such as outdoor urban landscapes. This complexity is increased by the constant movement and dynamic nature of both the observer and their environment in urban spaces. This paper presents a novel approach that integrates the capabilities of two foundation models, YOLOv8 and Mask2Former, as a pipeline to automatically annotate fixation points without requiring additional training or fine-tuning. Our pipeline leverages YOLO’s extensive training on the MS COCO dataset for object detection and Mask2Former’s training on the Cityscapes dataset for semantic segmentation. This integration not only streamlines the annotation process but also improves accuracy and consistency, ensuring reliable annotations, even in complex scenes with multiple objects side by side or at different depths. Validation through two experiments showcases its efficiency, achieving 89.05% accuracy in a controlled data collection and 81.50% accuracy in a real-world outdoor wayfinding scenario. With an average runtime per frame of 1.61 ± 0.35 s, our approach stands as a robust solution for automatic fixation annotation.

## 1. Introduction

Gaze behavior is widely acknowledged in the literature as a window into human cognition, making eye movement measurement a valuable tool in various fields seeking to understand human behavior (see [1] (pp. 2–9) for an overview). Despite its potential, the lack of efficient methods to automatically label recorded video footage has led researchers to either neglect the content-dependent aspect of the data (i.e., the objects viewed) or to manually label video frames in a more controlled and limited setting—a process that is both time- and labor-intensive and, therefore, does not scale well [2,3]. Automatic annotation of fixation points in eye-tracking videos is a central yet difficult task in mobile or remote eye-tracking research. This difficulty arises from the ever-changing and unpredictable nature of stimuli in urban environments, compounded by the continuous movement and dynamic interactions between the observer and their surroundings. This is especially true when the specific objects on which subjects focus are to be analyzed.

The advent of more advanced eye-tracking technologies has opened the door to extensive real-world studies, generating a vast number of data from naturalistic scenes, e.g., outdoor urban landscapes. Effectively annotating these videos holds immense potential for insights into human behavior in real-world settings. In recent decades, the need for the automatic annotation of eye-tracking videos has gained attention, and many attempts have been made to solve this problem [4,5,6]. The efforts to address this annotation challenge can primarily fall into two categories: traditional computer vision and image processing techniques, which suffer from limited scalability and are mostly suited for controlled laboratory environments (for example, they require a predefined set of objects or manual adjustments, which is impractical for processing a large number of data, which are easier to capture with today’s technology); and the more modern use of artificial intelligence and machine learning. Although the latter shows more promising results, the methods proposed up until now often require extensive training or fine-tuning and tend to simplify the task by limiting the number of objects for annotation, labeling the majority of gaze points as background [4].

**Figure 1:**
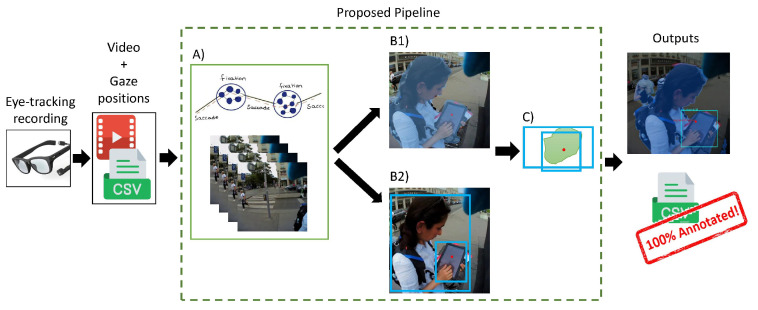
The pipeline begins by processing video and gaze data to detect fixations and extract the corresponding frames (A). These frames are analyzed for semantic segmentation and object detection (B1 and B2). YOLO identifies potential bounding boxes, which are then checked for mask coverage (C); the best-fitting box is selected as the final output. Results are visualized on the frame and detailed in a CSV file that includes labels, coverage metrics, and fixation data. This system ensures every fixation point is annotated and helps distinguish overlapping objects at different depths.

Here, we present our proposed pipeline (see Figure 1 and Section 3.3 for details) designed for automatic annotation of fixations in eye-tracking videos, integrating two powerful deep-learning approaches: object detection and semantic segmentation. Unlike current approaches, our goal is not only to ensure that every fixation point is potentially labeled but also to maximize the labels’ accuracy, thus pushing the boundaries of precision in fixation annotation. In developing our annotation pipeline for eye-tracking videos, we opted for a synergistic approach that takes advantage of the complementary strengths of object detection and semantic segmentation. While both methods can provide insights into the video data independently, their combined performance far exceeds their individual capabilities, increasing the accuracy and performance of our pipeline.

The motivation behind this combination stems from the distinct advantages that each method offers. Semantic segmentation performs well in extracting the precise boundaries of objects within an image, providing detailed masks that highlight the exact shape of items. This level of detail is invaluable, especially for detecting gaze shifts across adjacent objects, preventing misclassification when the gaze approaches the boundary of one object, thus ensuring accuracy. However, semantic segmentation’s limited ability to recognize a small set of object categories, without the ability to distinguish between multiple instances of the same category (for example, treating all cars in a scene as a single entity), poses challenges in complex scenes with a rich variety of elements. On the contrary, object detection excels at identifying and locating objects within an image by enclosing them in bounding boxes. This method is particularly effective for recognizing individual items, even when they are partially obscured or overlapped, making it ideal for analyzing scenes with multiple points of interest. By integrating these two approaches, our pipeline is designed to take advantage of the precise boundary detection of semantic segmentation and the broad and instance-wise object recognition capabilities of object detection. This combination ensures the comprehensive coverage and accurate annotation of all objects of interest within eye-tracking videos, facilitating a deeper and more detailed analysis of gaze behavior in diverse and dynamic environments.

We selected YOLOv8 [7] for object detection due to its speed, efficiency, and streamlined processing. Its training on the expansive MS COCO dataset [8], which includes 80 diverse classes of everyday items, provides an extensive reference for detecting a wide array of objects that are commonly encountered in daily scenarios. On the other hand, Mask2Former stands out for its precise detection of object outlines and its efficient inference capabilities among semantic segmentation models [9]. We deliberately chose the variant trained on the Cityscapes dataset [10] to provide our pipeline with the robustness required to handle the complexity of real-world urban environments.

We evaluated our pipeline’s performance, via random sampling, in two experiments, using manually annotated datasets as ground truth: in a controlled data collection experiment, where our method achieved an accuracy of 89.05%, and in eye-tracking footage recorded in a real-world wayfinding study, which achieved an accuracy of 81.50% compared to manually annotated datasets. This result underlines the great potential of our approach to advance eye-tracking research.

## 2. A Review of Automatic Annotation of Eye-Tracking Videos

Here, we address the various attempts in the literature to automate the annotation of eye-tracking videos. We divide our review into conventional approaches that use computer vision techniques and those that harness machine and deep learning. Specifically, when referring to computer vision techniques, we mean algorithms designed to detect a limited number of objects using image processing techniques. On the contrary, artificial intelligence-aided methods are trained on extensive datasets containing diverse objects, enabling them to also detect those objects in variations. In our review, we concentrate on the effectiveness and efficiency of methods, as far as they are reported by the authors. For effectiveness, this includes an evaluation of their capacity for detecting multiple object classes, their adaptability to various environments (be it indoor/laboratory settings or dynamic outdoor scenes), and their ability to deal with complications posed by proximate objects or those in varying depths. Regarding efficiency, our investigation is based on the information provided, focusing on computation time, resource consumption, and whether there is a requirement for the extra training or fine-tuning of models. Moreover, we have reviewed several papers that employ eye-tracking in the outdoor environment to answer their research questions and found that many either omit gaze annotation data due to its labor-intensive nature or undertake substantial manual annotation efforts. This underscores the vast research potential that could be unlocked with the availability of a pragmatically accurate and automated pipeline as an off-the-shelf product.

### 2.1. Computer-Vision-Aided Approaches

Previous attempts to overcome the tedious nature of manual video annotation were mainly based on computer vision techniques. For instance, Essig et al. [11] introduced JVideoGazer, a Java tool for automated video analysis, using Speeded-Up Robust Features (SURF) and the Fast Hessian Detector for the detection of objects and points of interest in videos. Evaluated with eye-tracking data from four participants interacting with objects of different sizes (a book, cookies, and a pack of tea), their system showed a significant reduction in annotation time, from more than an hour to 5–10 min per video of length 2–3 min. However, the requirement for manual object definition and challenges in predefining objects in real footage retained notable constraints.

Kurzhals et al. [6] approached the problem of video annotation from a different angle by assuming that there is a lot of redundancy in participants’ regions of interest (RoIs). They used clustering to identify and label common RoIs. This involved extracting a thumbnail around each gaze point and segmenting the video to group similar gaze points to reduce redundancy. Thumbnail similarity was assessed using SIFT features in a bag-of-features method and color histograms. However, since the method relies on the assumption of consistent RoIs and a standard window of 100 × 100 pixels (as the thumbnail), it reaches its limits in various real-world environments.

Netzel et al. [12] focused on incorporating scanpath and stimuli information into the annotation process rather than relying solely on AOI-based annotations, to support annotators with small or overlapping areas of interest (AOIs), as in subway maps. Their proposed software, which displays neighboring gaze points, improved the consistency of annotations and was tested by both novices and experts, achieving rates of 5 to 10.6 frames per minute. Despite its flexibility for different annotation tasks, the method is still time-consuming and dependent on human intervention, which limits the number of data that can be processed.

As an example of a limited object class, Paletta et al. [13] presented an approach for fully automated gaze detection on mobile devices for indoor and outdoor environments. Their method involves the use of a software toolbox, the Smartphone Eye Tracking Toolbox (SMET), which automates the annotation of eye-tracking videos by localizing the mobile display within the video frames using an adaptive computer vision technique. In outdoor tests with variable lighting, their approach achieved high accuracy in the localization of POR (Point of Regard) on mobile displays (approximately 1.5 ± 0.9 mm) with a very high recognition accuracy of 99.9%. Although such methods can perform excellently for a few objects, they cannot be applied to all objects in real experiments.

The list of works addressing the challenging task of automatically annotating videos using computer-vision-aided techniques goes on [14,15,16,17,18]. The main advantage of these works is the time saved in labeling the frames with various approaches that contribute differently to this efficiency. However, a common limitation is their restriction to either simple artificial visual stimuli with clearly defined static regions of interest or to a predetermined and limited set of object classes. Another drawback is that these methods are mainly semi-automatic, requiring human interference at different stages to obtain annotations. These limitations greatly restrict their applicability in real-world research scenarios. Efforts to address these key limitations have involved the adoption of artificial intelligence techniques.

### 2.2. Artificial-Intelligence-Aided Approaches

Recently, more papers have been published to address this problem using artificial intelligence techniques. A very recent work by Deane et al. [4] leveraged deep learning for video annotation, using Mask R-CNN [19]. Their system annotated a 20-s video in about 4.5 min, significantly faster than the 42 min required for manual annotation. However, it was limited to identifying only vehicles, people, and green spaces, with 77% of urban features labeled as “background”, which is not ideal for wayfinding and other studies, in which environmental objects such as buildings and traffic signs are of great importance.

To extend the object recognition categories, Uppal et al. [20] created the Multiple Object Eye-Tracking (MOET) dataset with images from screen-based videos of urban scenes (not from head-mounted eye-trackers). They trained Feature Pyramid Network (FPN) models [21] on MOET to localize attention focus through eye-tracking, also accounting for object depth. However, these FPN models did not perform as well as previously trained Faster R-CNN models [22], which exhibited superior integration of gaze and object recognition. The authors concluded that while such two-stage models are promising, improvements are needed.

To increase the accuracy of the annotation, Barz and Sonntag [23] improved visual attention detection with two methods, Image Classification (IC) and Object Detection (OD), using video and gaze data. IC classifies image patches around gaze points using ResNet, while OD uses Mask R-CNN to detect and prioritize objects that intersect with the gaze (by selecting the object with the highest prediction probability). On the VISUS dataset [24], both methods were successful on known classes, but they had problems with inaccurate eye-tracking and distinguishing between multiple instances of the same class. OD, however, better tolerated inaccuracies in the gaze due to bounding boxes. Two major limitations of their approach are that the dependency only on MS COCO in Mask R-CNN limits its use in different environments and that the maximum likelihood bounding box does not guarantee the correct detection of objects in some cases when objects are too close to each other or at different depths.

To compare different object recognition models, Kumari et al. [25] evaluated CNNs such as Faster R-CNN, YOLOv3, and YOLOv4 for object recognition in mobile eye-tracking data. They used the Lucas–Kanade method to interpolate eye-tracking in a laboratory study with students, which positively improved the overall results of all models. However, YOLOv4 performed best in terms of precision and speed, despite problems with overlapping shapes and irregular objects. They also reported tedious manual labeling, which required 12 h for a 7-min video, illustrating the intensity of the resource.

In summary, the application of AI in gaze annotation is promising yet underutilized. Current object recognition methods predominantly employ CNNs, yet the most comprehensive datasets for training object detection models, such as MS COCO and ImageNet, are deficient in outdoor environmental objects (e.g., buildings). The Mask R-CNN model, which performs instance segmentation by integrating aspects of both object detection (identifying the bounding box of individual objects) and semantic segmentation (categorizing each pixel without distinguishing between object instances), aligns closely with our method. Despite its efficiency and ease of training, Mask R-CNN’s utility is constrained by the specific classes included in its training dataset. Our method, while similar in integrating these computer vision tasks, differs by combining the strengths of two distinct models trained on separate datasets through the process of late fusion. Furthermore, while many existing systems offer their code on platforms like GitHub, they often are not ready for end users, requiring additional training or customization and generally covering a narrower spectrum of object classes than is available in the original training sets. They also face challenges in dealing with overlapping or closely-located objects. Our research aims to address these limitations.

### 2.3. Examples of Underutilized Potentials of Eye-Tracking Technology

Several studies have used eye-tracking and addressed the challenges of data annotation and analysis. For example, Kiefer et al. [26] analyzed tourists’ behavior in city panoramas and wayfinding tasks, using physical markers in the experiment environment for gaze detection, and noted the method’s scalability issues. In another study, Kiefer et al. [27] investigated participants’ navigational strategies by tracking their gaze during specific tasks. This was achieved by affixing markers to a handheld map, followed by manual data annotation. To lessen the burden of manual annotation, the authors recommended employing head-tracking technology alongside complex 3D environmental models. Simpson [28] studied gaze projection in outdoor settings using mobile eye-tracking, creating heat maps to represent the interaction of participants with their environment. The data were processed manually, which was time-consuming and error-prone. Simpson suggested that machine learning could significantly streamline this process. Dong et al. [29] investigated the differences in the visual behavior of pedestrians navigating in real-world environments compared to desktop environments. Despite using computer vision techniques to facilitate the automation of gaze annotations, they restricted the identified classes to “map” and “environment”, which includes all other objects. Flora Wenczel and von Stülpnagel [30] studied the impact of gaze behavior on landmark selection during navigation. Using a tedious and time-consuming manual frame-by-frame annotation in their experiments, they tried to understand how participants engage with visually distinct landmarks during wayfinding. The examples above illustrate scenarios where an automated method, such as the one presented in this paper, could offer significant advantages by providing consistent and fast results. Furthermore, the need for predefined markers or manual intervention limits the ability to repeat these experiments in more diverse or larger environments—a challenge that could be solved by an automated solution that does not require predetermined regions of interest. Additionally, manual annotations are not always consistent between different annotators. Depending on the accuracy of the eye-tracker, the fixation point could be located on the edge of two objects, and in such ambiguous cases, human annotators can assign it to either object. Here, the context of the fixations (before and after the fixation) becomes crucial [12].

Unlike manual processes, automated systems apply a uniform standard to each fixation point. For instance, when a fixation point falls on the last pixel of an object, the system consistently assigns it to that object, ensuring uniformity in annotation across multiple analyses. This level of consistency is a significant advantage, providing a reliable starting point for every fixation and establishing a baseline of consistent behavior. The presented system is designed and implemented to produce fast, consistent, and accurate annotations.

## 3. Methodology

To overcome the issues raised in the previous section, namely, the need for manual presets, the limited number of classes to be recognized, the challenges of overlapping objects, the training/fine-tuning required, etc., here we explain our approach for automatic fixation annotation, focusing on its core components: Mask2Former and YOLOv8.

### 3.1. Semantic Segmentation Component

Semantic segmentation, a fundamental task in computer vision, involves assigning each pixel of an image to a particular class. While traditional methods relied on Convolutional Neural Networks (CNNs), the advent of transformer-based models has fundamentally changed this field, especially by exploiting long-range spatial dependencies in images [31]. At the forefront of these transformer approaches is Mask2Former, developed by Facebook AI [32]. Mask2Former uses a transformer-based model, specifically the Swin transformer—a variant of the transformer architecture tailored for vision tasks that divides images into non-overlapping patches and processes them hierarchically [33]. Unlike standard CNNs, which typically use localized features, the Swin transformer uses both local and global attention mechanisms. This ensures that the model recognizes not only immediate but also distant patterns and relationships in images. Mask2Former’s efficiency is based on its unique “mask prediction” decoding method, which replaces the traditional pixel-by-pixel prediction of labels with a binary mask sequence, each representing different objects or semantic classes. This not only increases the flexibility of the model in complex visual contexts but also accelerates its processing speed [32]. As a foundation model, Mask2Former outperforms previous segmentation systems in terms of accuracy through its multiscale decoder and masked attention mechanism, and in terms of efficiency through its transformation decoder. Despite its sophisticated architecture, which makes fine-tuning difficult and requires significant computational resources for training, Mask2Former provides relatively fast inference by focusing on binary mask predictions rather than pixel-level details. The inference time for Mask2Former with Swin transformer is reported to be 84.5 ms on average (see Tables 2.9 and 3.3 in [34] for a speed comparison).

The variant of Mask2Former trained on the Cityscapes dataset is a suitable semantic segmentation for images of urban scenes. Cityscapes, a benchmark dataset, captures various urban scenes from several European cities and includes 30 unique classes, namely, street elements, vehicles, pedestrians, buildings, and more [10]. The complexity and richness of the Cityscapes dataset make it an ideal training ground for semantic segmentation models focused on urban environments. Training Mask2Former on Cityscapes not only increases its precision in understanding urban scenes but also extends its applicability in practical scenarios such as in the world eye-tracking. This combination of a transformer model architecture and a comprehensive training dataset sets Mask2Former (publicly available on Facebook AI’s GitHub repository [9]) apart from other semantic segmentation models for our intended purpose.

It is important to highlight that while Mask2Former is capable of detecting objects in the urban environment due to its training with the Cityscapes dataset, its scope is limited for many applications. In applications such as outdoor pedestrian wayfinding, which serves as a case study in this paper, the recognition of common context objects such as cell phones, tablets, and maps is critical. YOLO bridges this gap with its broader training on the MS COCO dataset.

### 3.2. YOLO (You Only Look Once) Component

Object recognition, another pillar of computer vision, involves the detection and localization of objects in images. While many methods have attempted to solve this problem, YOLO has emerged as the dominant solution, primarily because of its efficiency and accuracy [7,35]. Over time, YOLO has gone through several iterations, each refining its capabilities and resulting in the latest version: YOLOv8 [36]. Unlike conventional two-stage detectors that first identify and then classify regions of interest, YOLOv8 takes a unified approach. It divides an image into a grid and predicts multiple bounding boxes and class probabilities simultaneously for each grid cell. Its design revolves around a single forward pass through the network that enables real-time object detection—a critical requirement for applications that require instant responsiveness, such as real-time video analytics and autonomous driving.

YOLOv8’s power is achieved by its extensive training on the MS COCO dataset, a vast collection of over 300,000 labeled images spanning 80 object categories [8]. By immersing itself in this data-rich environment, YOLOv8 not only becomes familiar with a wide variety of object types but also gains the ability to recognize them in different contexts and lighting conditions. The result is a model that can quickly and accurately identify objects even in cluttered and dynamic scenes, which is essential in urban landscapes. Its efficiency combined with extensive training makes YOLOv8 an ideal choice for real-world applications that require fast and accurate object recognition. YOLOv8 has real-time processing capabilities, with inference times reported between 35 and 55 ms per image on an NVIDIA A100 GPU using TensorRT optimization [37] (also see Table 2 in [38] for a comparison of speeds between different object detection models). It can also process multiple images or video frames in a single batch, further speeding up the inference time. Several Python libraries offer the pre-trained YOLOv8 for object detection, and we used Ultralytics’ implementation here because it is extensively documented and easy to integrate into customized pipelines [36].

### 3.3. MYFix: Mask2Former-YOLO Fixation Annotation Pipeline

Here, we explain our approach (the code and data can be accessed via Geoinformation Resources Webpage.) to utilize the power of these two pre-trained models, YOLOv8 and Mask2Former, to annotate fixation points. Figure 1 shows an overview of the pipeline, which is explained in detail below. The strength of our pipeline is that we can effectively and efficiently annotate videos of urban scenes without having to deal with the complicated process of fine-tuning these advanced models.

**Figure 2:**
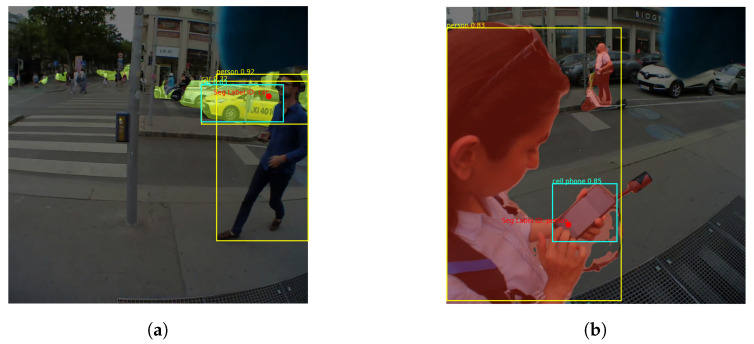
The depicted scenarios show two common cases where objects are positioned either at varying depths (**a**) or in close proximity to each other (**b**). In both cases, our method accurately selects the right box in cyan through the coverage check. Note that in (**b**), while the cell phone—a common context object—is wrongly detected as “person” by Mask2Former, it is correctly identified by YOLO (see text for more information on how a decision is made in case of disagreement). (**a**) There is a “car” mask detected by Mask2Former for the fixated object (depicted as a red dot). The mask and the fixation point are contained by multiple yellow boxes detected by YOLO since the car and person are at varying depths. (**b**) There is a “person” mask detected by Mask2Former for the fixated object (depicted as a red dot). Due to the proximity of the objects, the mask and the fixation point are contained in several yellow boxes.

As shown in Figure 1 the steps of the pipeline, for fully automated annotations, are outlined as follows:

**Step** **1:** **Prepare Input Data** In this initial step, the video recordings and associated gaze data are prepared by identifying the fixation points, as outlined in Figure 1, step *A*. We used the IDT (Identification by Dispersion-Threshold) algorithm by [39] as a commonly used dispersion-based algorithm to detect fixations (gaze-dispersion threshold: 0.02 deg; time threshold: 100 ms [40]). However, any other applicable fixation detection algorithm can be seamlessly integrated into this process, provided that the algorithm’s output conforms to the necessary format. The output is a CSV file containing some information including the fixation coordinates (normalized X and Y) along with the corresponding frame index from the video recording of the scene. Based on these indexes, the corresponding frames are then extracted from the video.
**Step** **2:** **Inference with the Backbone Models** The images are fed into the backbone models. Each image is processed simultaneously by Mask2Former and YOLOv8 (Figure 1, steps *B1* and *B2*). While the former retrieves the object masks, the latter identifies the bounding boxes around the detected objects. Then, the algorithm determines which mask and which bounding boxe(s) contain the fixation point.
**Step** **3:** **Mask Coverage Check** Since Mask2Former is designed to generate non-overlapping masks, each fixation point must be located within a single mask by definition. YOLO, on the other hand, can create overlapping bounding boxes, especially when multiple objects are nearby or located in depth. To overcome this challenge, our pipeline includes mask coverage checking (see Figure 1, step *C*): Of all the bounding boxes surrounding the fixation point, the one that has the greatest overlap (or coverage) with the selected mask is selected. As shown in Figure 2, this check ensures accurate annotations even if, as in Figure 2a, the fixation point is on a distant object (i.e., the car) that is overshadowed by a closer and larger object (i.e., the person), or as in Figure 2b, the detected objects are so close to each other (i.e., a person holding a cell phone). The mask coverage inside a bounding box is formulated as:
  Let the bounding box be defined by the coordinates $\left(x_{1},y_{1}\right)$ and $\left(x_{2},y_{2}\right)$. Let *M* represent the mask and *R* be the region of interest (ROI) extracted from the mask that is located within the bounding box. The coverage of the mask inside the bounding box is then given by:
  (1) $$C=\frac{\sum_{i,j\in R}M_{i,j}}{\left(x_{2}-x_{1}\right)\left(y_{2}-y_{1}\right)}$$
  *C* is calculated for all the candidate YOLO boxes, i.e., the boxes that contain both the fixation point and the mask, and then the box with the highest *C* value is selected as the final YOLO box/label.
**Step** **4:** **Generating the Output** The pipeline generates two primary outputs: segmented frames, each with labels, the detected mask, and the YOLO bounding box; and a CSV file. This file contains the frame index, the X and Y coordinates of the fixation, the labels identified by YOLO (if detected) and Mask2Former, and the calculated mask coverage. It also contains the selected YOLO bounding box and its corresponding confidence measure.

Through a fully automated approach, these steps assign at least one label to each fixation point, with the system outputting both labels if they exist. Typically when using eye-tracking data in research, the next step after obtaining annotated data is analysis. However, before analysis, the data need to be prepared. To illustrate an example of such preparations, we introduce some pre-processing guidelines essential for the examination of the labeled dataset. It is crucial to note that these guidelines are separate from the automatic annotations produced by the pipeline; rather, they function as our suggested pre-processing practices for conducting effective data analysis. The results of our analysis, stemming from these pre-processing steps, are presented in Section 5, and the corresponding code is also included with our pipeline.

Keeping the predicted labels from both models proves advantageous in preparing the data for the intended analysis. We propose the following pre-processing guidelines:

- Keep the label when both models agree.
- If one model fails to make a prediction, keep the prediction of the other: Mask2Former consistently generates a prediction; however, there may be cases where YOLO yields no prediction. In such cases, default to Mask2Former’s output.
- In general, users are encouraged to define a list of key objects relevant to their specific research objectives, recognizing that focusing on a narrower range of critical objects could enhance overall accuracy. In the event of disagreement:
  – Prioritize objects on the pre-defined list of interest. For example, as highlighted in Section 4, objects like *tablet* or *cell phone* are vital for studies on wayfinding due to their role as navigational aids. If the models’ predictions conflict and one (e.g., YOLO) identifies any of these essential categories, prioritize that output.
  – When predictions disagree without aligning with the priority list, consider making decisions based on mask coverage and YOLO’s confidence levels.

**Figure 3:**
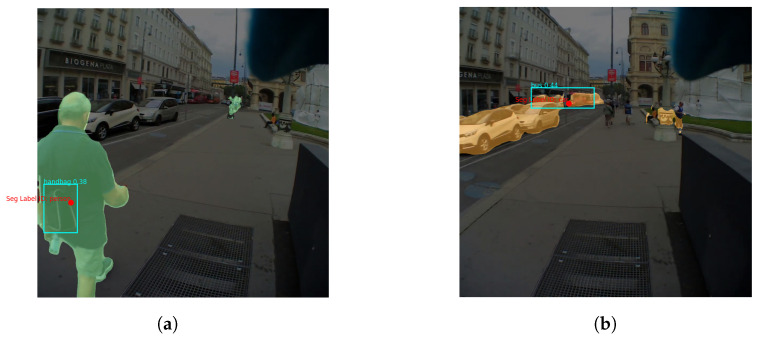
As an example, these images show the different semantic recognition levels of the two models and how beneficial this feature can be for some applications. (**a**) Mask2Former recognizes the person, but YOLO also recognizes the handbag the person is carrying. (**b**) Mask2Former recognizes a car queue, but YOLO correctly recognizes the bus in the queue.

An additional benefit of keeping both labels and our hybrid approach, in general, is the ability to uncover deeper semantic layers (see Figure 3); for example, Mask2Former might identify a *person* as the primary object, while YOLO can pinpoint specifics like a *handbag* being carried by that person (Figure 3a). Similarly, Mask2Former may categorize a line of vehicles as *car*, whereas YOLO distinguishes them as *bus* (Figure 3b). This specificity depends on the research aim and the importance of particular objects within that context.

It is also crucial to recognize that our existing pipeline does not account for the potential inaccuracies inherent in eye-tracking technology, which may not always place the gaze precisely on the intended location. When a fixation point is identified at the boundary of an object, our method deterministically assigns it to a single object. Integrating the context of the scanpath or employing fuzzy logic could help address these ambiguities, situations that typically necessitate the judgment of multiple human annotators to resolve.

## 4. Case Studies: Mobile Eye-Tracking in Urban Environments

In the following, we explain the two datasets we used to evaluate our approach (both studies strictly followed ethical guidelines: participants provided written informed consent for the use of their gaze data, and we maintained transparency of recording videos in public space using signage): first a 5.6-min outdoor gaze recording performed by the authors with a controlled setting to facilitate manual labeling of fixations; second, a practical application of eye-tracking videos recorded during an outdoor pedestrian navigation study. We deliberately chose this case study (outdoor navigation) because it contains extensive footage of real urban landscapes and its complexity makes it a challenging testing ground for our methodology.

For both datasets, a human annotator conducted manual labeling by examining the frames and identifying the objects of focus, which were marked on the frames with a red circle indicating the fixation point. These were labeled according to the same object classes utilized by the MYFix system. To facilitate this process, a custom-built Python application was used to sequentially present the images, allowing the annotator to assign a label to each one from this predefined list of labels. The outcome of this application is a CSV file with the frame indices and the labels selected by the annotator. This file was used to evaluate the output of the MYFix annotations.

### 4.1. Experiment 1: Controlled Data Collection

Throughout this article, we have highlighted the challenges of the manual annotation of gaze data, which is both time and labor-intensive. To facilitate this labor, we conducted a short experiment: we recorded 5.6 min of gaze data at a frequency of 200 Hz using PupilLabs Invisible glasses while walking down a central street in Vienna. The subject consciously focused on certain categories of objects, one after the other, for approximately the same amount of time. First, the subject’s gaze was focused exclusively (as much as possible) on buildings for one minute, then on vehicles, then on people, and finally on handheld devices such as a cell phone and a tablet (the devices were held in a natural way at different angles and orientations to simulate real-life use). Notably, while our method aimed to isolate each category to facilitate manual labeling, it was inevitable that the subject occasionally looked at unintended objects. Nevertheless, this targeted approach led to a significant reduction in manual labeling effort for all fixation points as we knew which object was of interest at any given minute.

This targeted fixation practice on handheld devices is particularly important for wayfinding research, where interactions with navigation aids such as maps or tablets/cell phones are an important component of navigation behavior. Current eye-tracking software solutions are equipped with surface detection capabilities that can localize eye contact with predefined surfaces. However, these methods rely heavily on image processing and require visible markers in the image to track gaze relative to these surfaces. The Surface Tracker plugin in Pupil Player v3.5.1 [41], for example, requires the post-processing of recordings to identify markers, define and edit surfaces, and generate exports of gaze visualizations within these defined areas. In this first experiment, we wanted to find out how accurately our method labels fixations on objects that appear in the visual urban landscape and whether our approach is also suitable for finding interactions with such navigation devices without post-processing and additional effort.

The 5.6-min recording with a frame rate of 30 frames/second provided 10,080 images. However, the IDT algorithm with the specified threshold values recognized 2522 fixation points. Therefore, the system ended up having to process 2522 images, which took 58.87 min on an Alienware system using sequential processing (parallel processing would reduce this time to the number of cores for parallelization, but we intentionally ran sequential processing to obtain the individual runtime estimate per frame).

### 4.2. Experiment 2: Pedestrian Wayfinding in Outdoor Environments

Since the data collection from the first experiment was controlled, the number of gaze switches between different objects in successive frames was limited. To evaluate the system using a dataset from real participants, we used a very small portion of gaze data obtained from a past in situ wayfinding study. The reason for using only a small portion is simply based on the previously mentioned fact that creating the ground truth for validation would demand extensive manual effort. In this study, 56 people ($mean_{age}$ = 31.16 years, $std_{age}$ = 5.93), comprising 22 females and 34 males, navigated through different routes in the city center of Vienna while their eye movements were recorded via the same PupilLabs Invisible glasses. The participants were equipped with a tablet device that showed them a customized map of the city. For each route, they were asked to first locate themselves and determine their location on the map (requiring many visual interactions with the tablet device and the environment); then, they were shown a destination on the map and asked to draw a preferred route to walk to that destination (here most of the visual attention was on the tablet device), then they had to walk to the destination using their normal navigation behavior as if they were alone (during this step although they were allowed to use the digital map as many times as they wanted; the main visual attention was to the surrounding environment). At the beginning of the experiment, they also had to look at information that was visualized on a laptop and a cell phone. This setup provided a context-rich dataset to test our approach as it includes not only the urban environment but also some everyday objects (tablet, cell phone, etc.), which emphasizes the importance of the combined approach we proposed.

Approximately 30 min of video footage was recorded from each participant during each trial, accumulating a considerable number of data. For the further evaluation of our proposed system, we randomly selected a video with a length of 24 min and 15 s (43,648 frames) from this large collection to serve as a test case. This video happened to be recorded near a famous tourist spot in Vienna providing a wide field of view of the surrounding area. This provides practical test data as many features of the urban environment need to be recognized there. The IDT algorithm detected 11,694 fixations for this recording. We sequentially processed a subset of these frames since, as previously mentioned, manually labeling so many images would not be practical. This subset was randomly selected by choosing 10% of the whole set (rounded up to 1200 frames). The fully automatic annotation on the same Alienware machine took 31 min (manual labeling took approximately 5.5 h). The accuracy of our approach for both experiments is discussed in detail in Section 5.

### 4.3. Comparative Overview of Experimental Environments

While comparing the two environments used in our experiments, differences emerge that could potentially impact the annotation process and performance. Experiment 1 took place in Gusshausstrasse, Vienna, a tertiary road with one lane for passing cars. In this setting, the subject deliberately focused on specific object categories, leading to reduced unintended gaze shifts.

Experiment 2 occurred at a crossing near the Opera House in Vienna, located along a secondary road with multiple tram lines; buses; and over four lanes for cars. The outdoor environment presented a broader field of view and a higher density of dynamic objects. The natural movement of the participants also led to increased motion blur, complicating the annotation process. These differences underscore the importance of assessing the system performance under varied environmental conditions to ensure robustness and reliability in real-world applications.

## 5. Results

In this section, we report the runtime and evaluation metrics for our method, including accuracy, precision, recall, and F1 score. The interpretation of these results is presented in Section 6. The comparison is based on the selected label (according to the provided guide in Section 3.3) and the manual labels. *Accuracy* is computed as the ratio of the total number of correct predictions to the total number of input samples, indicating how often the model is correct across all classes. *Precision* is the ratio of correctly predicted observations of a class to the total predicted observations of that class, showing how many of the predictions made by the model for a class are actually correct. *Recall*, also known as *sensitivity*, is the ratio of correctly predicted observations of a class to all observations in that class, indicating how well the model captures all examples of a class. *F1-Score* is the harmonic mean of precision and recall and helps us to understand how precise and reliable the model is.

Given the presence of class imbalance in our dataset, we present both weighted and macro averages for our metrics to reflect this imbalance appropriately. The *Macro Average* is calculated by averaging the performance of each class without considering its frequency in the dataset so that each class is treated equally regardless of any imbalance. The *Weighted Average*, on the other hand, takes into account the frequency of each class (class support) when calculating the average metric, thus reflecting the unequal distribution of classes. These metrics provide information on the overall effectiveness of the models but also on their performance per class, taking into account the potential imbalances in the dataset.

In analyzing the results, two key points need to be considered. First, the MS COCO dataset lacks a *tablet* category. Among different digital screens, it only contains labels for *tv*, *laptop*, and *cell phone*. We can consider it pragmatically acceptable if *tablets* were consistently mislabeled as another device, such as a *cell phone*. This applies to any other unlisted object across the models utilized. Second, we observe a distinction in how Mask2Former and YOLO categorize certain entities: Mask2Former differentiates between *rider* and *person*, as well as *road* and *sidewalk*, while YOLO differentiates *car* from *truck*. To enhance the utility of our findings, we merged related categories into broader classes: *sidewalk/road*, *person/rider*, *car/truck*, and *tablet/cell phone*. This decision was informed by our research focus on wayfinding, where distinguishing between such categories may not critically impact our objectives, illustrating our tailored approach to label merging based on specific research needs. Merged labels represent a higher semantic abstraction, combining related categories into broader classes, while unmerged labels retain the original categories without combining them.

**Figure 4:**
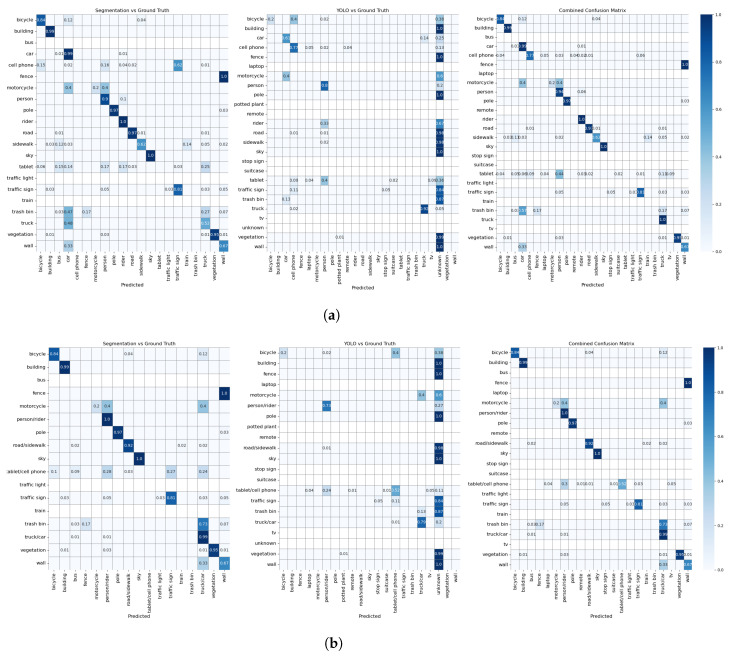
Confusion matrices of Exp.1: As can be seen in both tests, the combined approach has a better detection performance. (**a**) Confusion matrices of Exp. 1 with unmerged labels. (**b**) Confusion matrices of Exp. 1 with merged labels.

**Figure 5:**
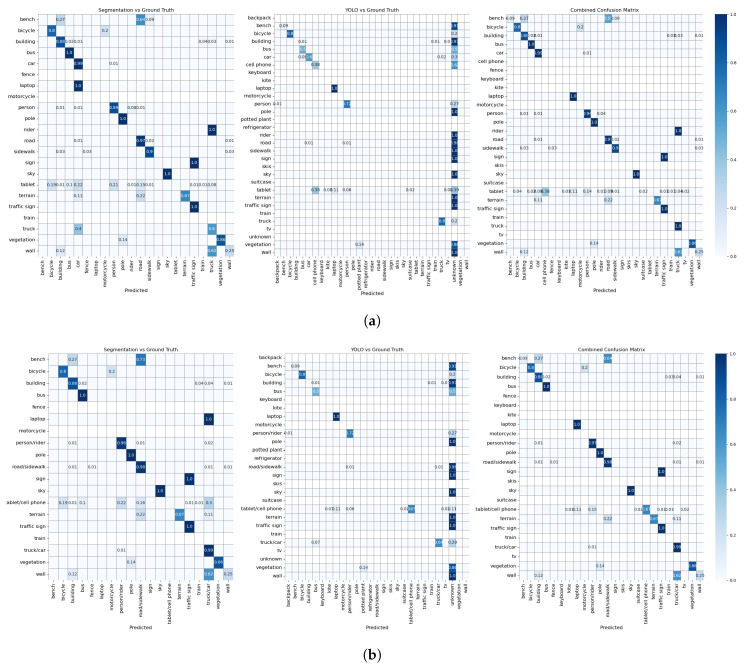
Confusion matrices of Exp.2: As can be seen in both tests, the combined approach has a better detection performance. (**a**) Confusion matrices of Exp. 2 with unmerged labels. (**b**) Confusion matrices of Exp. 2 with merged labels.

Table 1 shows the performance metrics, with the best performances belonging to the experiment with the combined model and merged labels, while Figure 4 and Figure 5 show the confusion matrices for each experimental condition in Exp 1 and 2, respectively. For clarity, the values of the confusion matrix are normalized (from 0 to 1), with cells representing a zero value left blank to provide a comprehensive overview of the robustness of our approach and the strategic reasoning behind our label merging decisions.

**Table 1:** Performance metrics of YOLO, Mask2Former, and our Combined Approach (MYFix) on the two experimental datasets. The metrics include Accuracy (Acc), Precision (Prec), Recall (Rec), and F1-Score (F1-S), under conditions with unmerged and merged class labels. The results are presented with both macro and weighted averages to account for class distribution. *Support* indicates the number of frames evaluated in each experimental condition. As highlighted in the table, the best performance was achieved with MYFix.

				Exp1		Exp2	
				Support = 2522		Support = 1200	
				*Unmerged Label*	*Merged Label*	*Unmerged Label*	*Merged Label*
***Models***	***YOLO***	***Acc***		0.132	0.152	0.116	0.207
		***Macro Avg***	***Prec***	0.66	0.63	0.52	0.50
			***Rec***	0.41	0.43	0.53	0.56
			***F1-S***	0.12	0.10	0.12	0.13
		***Weighted Avg***	***Prec***	0.95	0.95	0.97	0.97
			***Rec***	0.13	0.15	0.12	0.21
			***F1-S***	0.13	0.18	0.13	0.26
	***Mask2Former***	***Acc***		0.756	0.768	0.690	0.703
		***Macro Avg***	***Prec***	0.57	0.57	0.55	0.59
			***Rec***	0.69	0.73	0.67	0.70
			***F1-S***	0.48	0.51	0.44	0.41
		***Weighted Avg***	***Prec***	0.91	0.91	0.86	0.86
			***Rec***	0.76	0.77	0.69	0.70
			***F1-S***	0.72	0.73	0.64	0.64
	***Combined Approach (MYFix)***	***Acc***		0.82	***0.890***	0.70	***0.815***
		***Macro Avg***	***Prec***	0.59	***0.57***	0.53	***0.58***
			***Rec***	0.53	***0.49***	0.49	***0.52***
			***F1-S***	0.54	***0.50***	0.48	***0.51***
		***Weighted Avg***	***Prec***	0.84	***0.96***	0.74	***0.98***
			***Rec***	0.82	***0.89***	0.70	***0.81***
			***F1-S***	0.83	***0.87***	0.72	***0.85***

To ensure scalability and effectiveness for large datasets, the runtime efficiency of each component within our system was evaluated. The average inference time for Mask2Former was found to be 0.950±0.056 s per image, while YOLO had a significantly higher processing speed with an average of 0.332±0.143 s per image. Our measured time for both models is in line with the review by [42]. In addition, the mask coverage check and writing of the annotated frame to disk required an average of 0.493±0.015 s per image, resulting in an average total execution time of 1.608±0.355 s per image. These values illustrate the computational effort of the individual processes per image. For larger data sets, the total computation time can be significantly reduced by using a parallelized execution mode. The amount of time savings correlates directly with the number of cores used for parallelization, highlighting the potential for significant efficiency gains when processing large image collections.

## 6. Discussion

Here, we presented our approach for automatic annotation of fixation points from eye-tracking videos and evaluated it with recordings in urban outdoor areas. From the results presented in Table 1, we can see several trends and performance differences between the models and post-processing approaches applied to the two datasets. First and foremost, unlike most approaches [4,13], our approach provided potential labels for all fixation points.

Although we have provided both macro and weighted averages to show the inherent nature of imbalance classes and their impact on the evaluation matrix, we discuss the weighted results here as we want to account for the imbalance classes. Table 1 shows that YOLO has high precision but low recall in both experiments, suggesting that it makes very accurate predictions for the classes it recognizes but fails to identify many instances of other classes. This behavior is consistent with the training of YOLO, which is not on images of outdoor environments; therefore, it tends to be cautious and often only correctly identifies known objects. Mask2Former shows a similar trend, although not as pronounced as YOLO. In both experiments, precision exceeds recall, suggesting that while the model reliably labels known instances of a class, it misses numerous other instances. This property is also consistent with the design of the model, which is not trained on everyday objects. The confusion matrices (Figure 4 and Figure 5) confirm these observations and show that each model recognizes objects within its knowledge domain well but performs poorly for unknown objects. These results confirm our considerations regarding the selection of these specific models.

The combined approach, shown in Table 1, not only outperforms accuracy but also achieves a more balanced relationship between precision and recall, resulting in an F1 score that more closely aligns with these metrics. This combined model, as we aimed for, takes advantage of the strengths of the two individual models and delivers consistent performance across different classes. Furthermore, applying our merging technique has been shown to improve results by shifting the focus of our system to broader semantic categories that are more relevant to the research objectives at hand. In particular, for the non-existing *tablet* class, merging the label with *cell phone*, i.e., selecting another semantic level of detail, significantly reduces the number of incorrect predictions.

**Figure 6:**
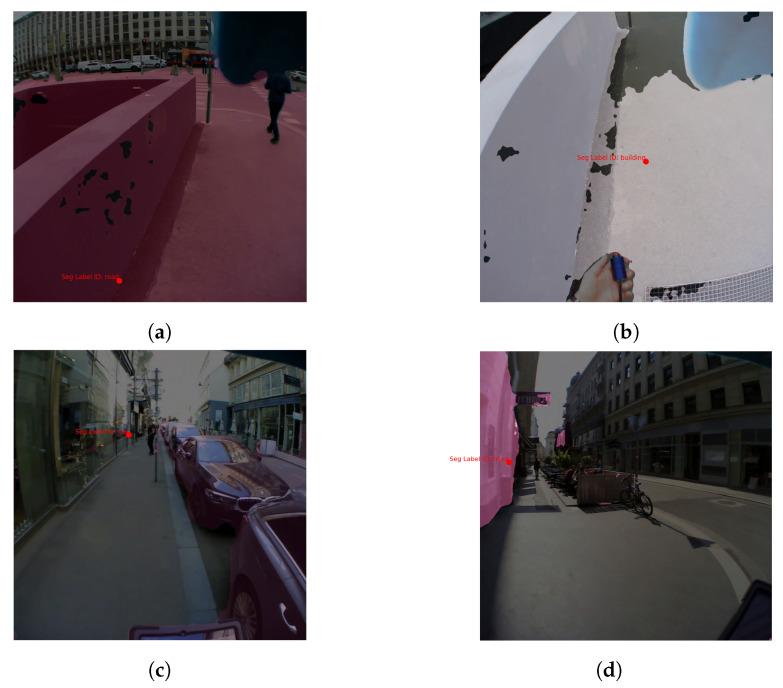
Examples where Mask2Former incorrectly identifies objects, mainly due to their atypical placement in the image compared to the model’s training data. (**a**) The object is correctly detected as a road. (**b**) Due to the head orientation (looking down), the same object is detected as a building. (**c**) The reflection of a car in the glass window of a building is detected as a car. (**d**) Building is confused with truck because of its orientation in the image.

Mask2Former shows a drop in performance from Exp. 1 to Exp. 2, which can be due to several factors, such as the higher complexity of the environment in terms of more dynamic objects in the broader field of view as explained in Section 4.3. We looked closely at the images our model labeled incorrectly and noticed some common mistakes. For instance, as shown in Figure 6, Mask2Former often gets mixed up in the second experiment. Take Figure 6a, where it identifies the *road* correctly, but when the view shifts to the sidewalk and the buildings up against the sky disappear from the frame, the model mistakes the same area for a *building* (Figure 6b). Often in our test frames, Mask2Former wrongly labels a *road* or *sidewalk* as a *building* when the participant’s head is rotated towards the ground. There are also cases when crossing an intersection and looking towards the buildings on the side; here, Mask2Former confuses the *building* with a *truck* or a *train*, probably because the buildings are not placed as they were in its training images. Another interesting case is when reflections in buildings’ glass windows throw off Mask2Former, as seen in Figure 6c.

Figure 4 shows the three confusion matrices corresponding to the predictions of the different models compared to the manual annotations. When interpreting these, it is important to keep in mind that Mask2Former and Yolo v8 have different class lists. In the Mask2Former matrices in both Figure 4a,b, we see strong diagonal elements for classes such as *person/rider* (1.0), *sky* (1.0), *building* (0.99), *car/truck* (0.99), *pole* (0.97), *vegetation* (0.95), *road/sidewalk* (0.92), and *traffic signs* (0.81), indicating a very good performance for these classes. The second matrix shows the performance of YOLO. Here, the classes *cellphone*, *person/rider*, and *car/truck* show strong diagonal values, which indicates that YOLO recognizes these objects particularly well. The combined confusion matrix, which aggregates the results of both models following the pre-processing guidelines presented in Section 3.3, outperforms both individual models, which is in line with the findings of [23].

As shown in Figure 5, the corresponding confusion matrices of the second dataset, Mask2Former, again have very high values in some classes, including *bus* (1. 0), *pole* (1. 0), *sky* (1. 0), *traffic sign* (1.0), *car/truck* (0.99), *vegetation* (0.86), *person/rider* (0.96), *road/sidewalk* (0.98), and *building* (0.88), which indicates accurate predictions. YOLO is very accurate in recognizing *laptop* (1.0), *bicycle* (0.80), *person/rider* (0.72), and *tablet/cell phone* (0.67), which is a considerable improvement due to the merging of the labels. 

It is important to know the strength of the models at correctly recognizing certain objects, but it is also equally important to know when they fail and which objects confuse them. Off-diagonal elements in the confusion matrices in Figure 4 and Figure 5 represent misclassifications—cases where the model was confused. We discuss the off-diagonals of each matrix only with respect to the classes the model could potentially detect (the class had been part of its training dataset). For instance, as Mask2Former is not trained on *cell phone*, *tablet*, *trash bin*, and *laptop* we do not consider the misclassifications of these classes by Mask2Former. The same applies for YOLO with respect to the *building*, *pole*, *road/sidewalk*, *terrain*, *traffic light*, *vegetation*, and *wall* classes.

Examining the non-diagonal elements of the Mask2Former confusion matrices from Exp. 1, it is evident that the model often confuses *motorcycle* with *rider* and *car/truck*. The difficulty arises when the fixated object is distant in the image, a challenge similar to shape irregularity issues previously noted by [25]. To simplify vehicle recognition, one could group all vehicles and their drivers under a general *vehicle* category, a strategy adopted in other studies as well [11,13,25]. Our pipeline allows for such categorization flexibility during the data analysis preparation stage. Furthermore, the mislabeling of *wall* suggests the model’s training lacked diversity in objects typically categorized as walls.

**Figure 7:**
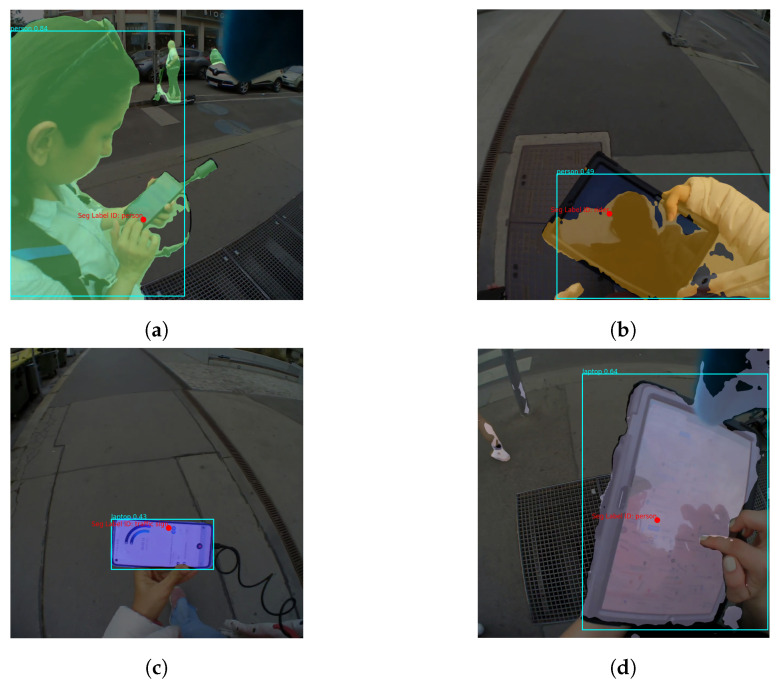
Example frames of cases where YOLO confuses handheld devices. (**a**) A cell phone held by the experimenter causes YOLO to confuse the person holding the phone with the phone, especially if the fixation point is at the edge of these two objects. (**b**) The reflection of the person on the black screen of the tablet due to the lighting conditions confuses YOLO and leads to incorrectly recognizing the person. (**c**) Cell phone held in landscape orientation is recognized as a laptop by YOLO. (**d**) A 12.4-inch tablet device showing a map to the user is misclassified as a laptop by YOLO.

A look at the misclassified samples of *bicycle* from YOLO shows that they were either not recognized or confused with *cell phone* or *cell phone/tablet*. There were only a few instances of *bicycles* in the collected data, and they were all located very far away from the observer. The same applies to *motorcycles* that were misclassified as *car/truck* or not recognized at all. The same pre-processing approach can be taken if a higher level of semantics can fulfill the requirements of the experiment. There is one particularly pronounced misclassification: several instances of class *tablet* are either not recognized at all or labeled as *person/rider*. A manual inspection of these cases revealed that the reason for most misclassifications is the local lighting conditions. As can be seen in Figure 7b, the tablet surface is black and the reflection of the person carrying the tablet can be seen on the surface, and since one/both hand(s) are usually also visible, the combination is recognized as human. This also applies to Mask2Former in Exp. 1. However, the weather was cloudy when Exp. 2 was recorded. As a consequence, the content of the tablet displaying a custom map was visible in the frames, causing YOLO to recognize *tablet* mainly as *cell phone* or *laptop*.

Similarly, in Exp. 2 the *cell phone* was often mistaken for a *person/driver* and not a *tablet*. However, the reason this time was that the device was held by the experimenter rather than the observer, making the experimenter’s figure fully visible in the scene (see Figure 7a). Furthermore, despite the merging of the labels *tablet* and *cell phone*, 11% of the tablets were still mislabeled as *laptops*, especially when oriented in portrait mode (like a laptop), as shown in Figure 7d. This confusion extends to cell phones in landscape mode from Exp. 1 (Figure 7c).

## 7. Conclusions and Future Work

In this paper, we presented a pipeline for automatic fixation point annotation using two pre-trained foundation models. We have provided empirical evidence that this combined approach is suitable for annotations in complex and dynamic outdoor urban environments. The main features of our approach are: first, it provides a potential label for all fixated objects (no background); second, it can effectively detect closely spaced or overlapping objects (different depths) due to the combined performance of the models used together with mask coverage verification; third, it provides high accuracy, especially when a more abstract semantic level is sufficient (merging labels to more abstract semantics); and fourth, the computation time and required resources are quite low due to the fast inference performance of the models used.

Overall, our results suggest that a combined approach is beneficial when dealing with complex datasets, especially when the goal is to obtain potentially accurate labels for all fixation points. We have compared the performance of our approach in two different scenarios, although we acknowledge the absence of comparison against existing methods. To the best of our knowledge, the existing methods use different approaches that result in most environment objects being annotated as background. Therefore, a direct comparison with our method would not be suitable.

While our findings are promising and offer valuable insights, there are areas for future improvement. Advanced segmentation models could refine mask edges and identify a broader range of surfaces, potentially enhancing performance, especially when trained on datasets like Cityscape. Additionally, customizing YOLO to a predefined set of labels relevant to specific tasks, such as outdoor wayfinding, could notably enhance recognition accuracy by excluding irrelevant object classes. Since using the context of fixations, especially the associated semantics, has shown promising results in gaze prediction research, our pipeline could be extended to consider the context for each fixation and be able to predict gaze points. Finally, our experience with outdoor data collection suggests the use of non-reflective stickers on the screen of electronic devices to improve their recognizability. These improvements underline the significant progress made so far and the further potential of this research.

## Data Availability

The code and sample data used in this paper can be accessed via the webpage https://geoinfo.geo.tuwien.ac.at/resources/ (Geoinformation Resources Webpage, accessed on 26 February 2024).
